# Characteristics, pathogenic and therapeutic role of gut microbiota in immunoglobulin A nephropathy

**DOI:** 10.3389/fimmu.2025.1438683

**Published:** 2025-02-06

**Authors:** Kaijin Yao, Lingqian Zheng, Wenmin Chen, Yina Xie, Chunling Liao, Tianbiao Zhou

**Affiliations:** Department of Nephrology, The Second Affiliated Hospital of Shantou University Medical College, Shantou, China

**Keywords:** immunoglobulin A nephropathy, gut microbiota, dysbiosis, immune responses, modulate

## Abstract

Immunoglobulin A nephropathy (IgAN) is the most prevalent glomerulonephritis in the world, and it is one of the leading causes of end-stage kidney disease. It is now believed that the pathogenesis of IgAN is the mesangial deposition of immune complex containing galactose-deficient IgA1, resulting in glomerular injury. Current treatments for IgAN include supportive care and immunosuppressive therapy. A growing number of studies found that the gut microbiota in IgAN was dysregulated. Gut microbiota may be involved in the development and progression of IgAN through three main aspects: destruction of intestinal barrier, changes in metabolites and abnormal mucosal immunity. Interestingly, therapies by modulating the gut microbiota, such as fecal microbiota transplantation, antibiotic treatment, probiotic treatment, Chinese herbal medicine Zhen Wu Tang treatment, gluten-free diet, and hydroxychloroquine treatment, can improve IgAN. In this review, the alteration of gut microbiota in IgAN, potential pathogenic roles of gut microbiota on IgAN and potential approaches to treat IgAN by modulating the gut microbiota are summarized.

## Introduction

Immunoglobulin A nephropathy (IgAN) is the most common form of glomerulonephritis in the world, characterized by the deposition of IgA-dominated immune complexes in the glomerular mesangium (1). The prevalence of IgAN varies in geography, with the Asia-Pacific region having the highest prevalence and Africa the lowest (1). It is reported that the overall population incidence of IgAN is at least 2.5 per 100000 (2). IgAN accounts for the largest proportion of causes of chronic kidney disease (CKD) in China (3). The clinical manifestations of IgAN are varied, with the most common manifestations being asymptomatic hematuria and progressive kidney disease (4). It is now believed that four major hits are involved in the pathogenesis of IgAN. Firstly, circulating galactose-deficient IgA1(Gd-IgA1) increases hereditarily; secondly, the autoimmune antibodies IgG and IgA directed against Gd-IgA1 are synthesized to form immune complexes; finally, the immune complexes containing Gd-IgA1 are deposited in the glomerular mesangium, which activates the mesangial cells and the complement system, leading to glomerular injury (5). 30% to 40% of patients with IgAN will develop end-stage renal disease (ESRD) within 20 to 30 years after the diagnosis (6). The current treatment of IgAN mainly includes supportive care and immunosuppressive therapy (7). Further, supportive care mainly includes control of blood pressure and proteinuria and correction of diet and lifestyle, while immunosuppressive therapy mainly includes the use of corticosteroids (8). All IgAN patients are recommended to receive supportive care, while immunosuppressive therapy is recommended for patients whose proteinuria is not effectively controlled after receiving maximal supportive care (8). However, immunosuppressive therapy with corticosteroids is still controversial due to its efficacy and side effects (9).

Gut microbiota, a highly complex community of microbes in the gastrointestinal tract, contains over 1,000 different species of bacteria (10, 11). It coexists and interacts with the host, and plays an important role in substance metabolism, maintaining the integrity of intestinal mucosa, defending against pathogens and immune regulation (12). In recent years, crosstalk between the gut and kidney, also known as the gut-kidney axis, has been found in some kidney diseases (13). For example, in CKD, uremia has an influence on the composition and metabolism of the gut microbiota, while the metabolism of gut microbiota produces important uremic toxins (14). It was also found that the gut-kidney axis plays an important role in IgAN (15). Gut microbiota might be involved in the development and progression of IgAN (16). Interestingly, some treatments that modulate the gut microbiota have been shown to improve IgAN (17). In this review, we summarize the changes of gut microbiota in IgAN, the potential pathogenic role of gut microbiota in IgAN, and the potential therapies of IgAN by modulating gut microbiota.

## The alteration of gut microbiota in IgAN animal models

In recent years, some studies have found that the gut microbiota of IgAN animal model was changed compared with that of normal control animals. Li et al. (18) found that IgAN rats had different diversity and composition of gut microbiota from normal rats. Moreover, they showed that the blood creatinine and urine protein were closely related to the changes of gut microbiota in IgAN rats (18). And some biochemical indices such as blood creatinine, blood urea nitrogen, urine volume, 24-h protein urine, etc., were positively or negatively correlated with the abundance of several genera of gut microbiota (18). These suggested that there is a potential association between gut microbiota dysbiosis and renal insufficiency. Likewise, IgAN mice also had the gut microbiota with different diversity and composition compared with normal control mice. These mice showed gut microbiota dysbiosis with a significant reduction in *Bifidobacterium* levels, which can also be found in IgAN patients (19). Studies comparing gut microbiota between IgAN animal models and normal animal controls are summarized in **Table 1**.

**Table 1:** The alteration of gut microbiota in IgAN animal models.

Study(Year)	Animal model	The alteration of gut microbiota	Correlation	Intervention outcome	Reference (PMID)
Li, et al. (2021) (18)	IgAN rats	**Phylum:** Cyanobacteria and Euryarchaeota ↓	Serum levels of blood urea nitrogen and creatinine, 24-h protein urine were positively or negatively associated with the richness of several genera.	Treatment of Zhen Wu Tang alleviated gut microbiota dysbiosis and attenuated kidney damage.	33584283
		**Order:** Betaproteobacteriales ↑; Methanobacteriales↓			
		**Family:** Lachnospiraceae and Burkholderiaceae↑; Muribaculaceae and Christensenellaceae↓			
Tan, et al. (2022) (19)	IgAN mice	**Genus:** Bifidobacterium and Lactobacillus↓; Helicobacter and Alloprevotella↑	**-**	Probiotics supplementation improved gut microbiota dysbiosis. Both probiotics and their SCFA metabolites could attenuate the clinicopathological manifestations of IgAN by blunting NLRP3 signaling.	36038927

↑, higher abundance; ↓, lower abundance.

In conclusion, IgAN animal models exhibit gut microbiota dysbiosis. The difference of gut microbiota is expected to be a biomarker for the non-invasive diagnosis of IgAN.

## The alteration of gut microbiota in IgAN patients

**Figure 1:**
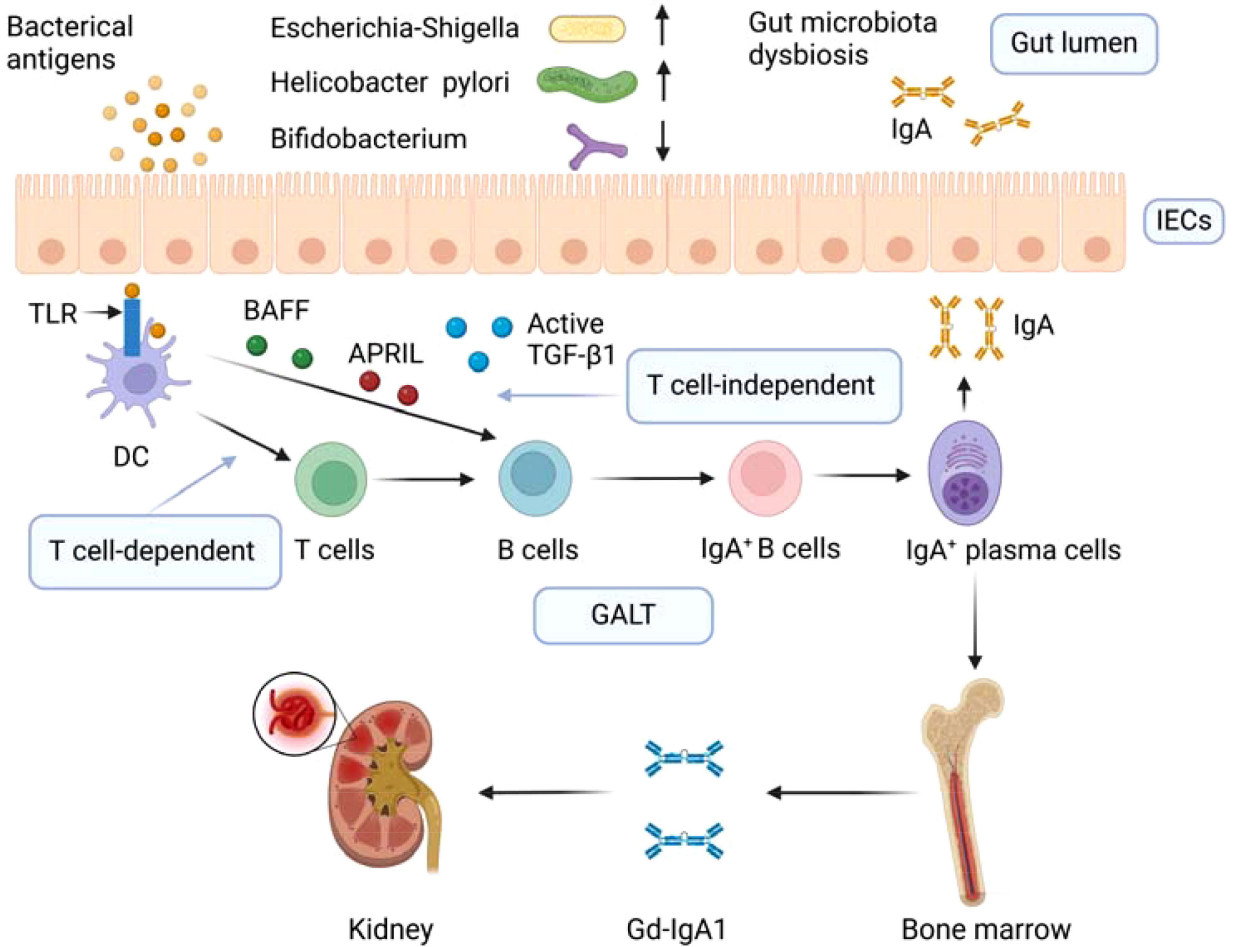
Gut microbiota dysbiosis and aberrant mucosal immune response in IgAN. Dysbiosis of gut microbiota in IgAN is manifested by a decrease in beneficial bacteria such as Bifidobacterium and an increase in harmful bacteria such as Escherichia-Shigella and Helicobacter pylori. Gut microbiota dysbiosis leads to intestinal microbial infection. Intestinal microbial infections boost the class switch of naive B lymphocytes to IgA antibody-secreting cells by T cell-independent and T cell-dependent pathways in GALT. In the T cell-dependent pathway, dendritic cells express TLR that recognizes microbial antigens and thus activating T cells, which promotes the differentiation of B cells into IgA+ B cells. In the T cell-independent pathway, microbial antigens can promote the release of BAFF and APRIL, and active TGF-β1, BAFF, and APRIL promote the preferential switching of TLR-activated B cells from IgM to IgA. IgA+ B cells differentiate into IgA+ plasma cells. Due to abnormal homing, lymphoplasmic cells producing mucosal IgA1 gradually migrate from mucosal lymphoid tissue to bone marrow, resulting in excessive production of mucosal IgA1 in the systemic circulation. These overproduced IgA are galactose-deficient IgA1, and the immune complex containing galactose-deficient IgA1 is deposited in the mesangium leading to glomerular damage. IECs, intestinal epithelial cells; DC, dendritic cell; TLR, Toll-like receptor; BAFF, B cell activating factor of the tumor necrosis factor family; APRIL, a proliferation-inducing ligand; TGF-β1, transforming growth factor β1; GALT, gut-associated lymphoid tissue; Gd-IgA1, galactose-deficient IgA1. Created with BioRender.com.

Similarly, increasing studies also have revealed that IgAN patients have different gut microbiota from normal people (20–23). Furthermore, IgAN patients have a different diversity of gut microbiota from normal people, and the diversity of gut microbiota in IgAN patients is lower than normal people (19, 24, 25). It was also found that the composition of gut microbiota in IgAN patients was significantly different from that in normal controls (19, 25–31). Moreover, the dysbiosis of gut microbiota in IgAN patients also differs between men and women (32). Most studies compared the gut microbiota of IgAN patients with normal controls and found the consistent result that the relative abundance of *Escherichia-Shigella* increased in the gut microbiota of IgAN patients (22–24, 26, 33–36). Zhong et al. (26) reported that patients with a higher urine RBC count and more severe proteinuria showed a higher abundance of *Escherichia-Shigella*. Furthermore, it was reported that *Escherichia-Shigella* was positively correlated with the urinary albumin-to-creatinine ratio and urine red blood cells account, but was negatively correlated with the estimated glomerular filtration rate (eGFR) (23, 33, 35). Interestingly, Zhao et al. (24) found that the abundance of *Escherichia-Shigella* in patients who received immunosuppressive therapy and achieved clinical remission returned to be similar to that in normal controls. *Helicobacter pylori* (*H. pylori*) infection is an important factor in gastric mucosal injury, and about 1% of patients with *H. pylori* infection will develop into gastric cancer (37). Liu et al. (38) found that the infection rate of type I *H. pylori* in IgAN patients was significantly higher than that in normal controls. Blood pressure, proteinuria and blood urea nitrogen levels in IgAN patients with *H. pylori* infection were significantly higher than those in uninfected patients, especially in patients with the infection of type I *H. pylori* (38). Moreover, IgAN patients with type I *H. pylori* infection had higher plasma Gd-IgA1 levels compared with uninfected patients (38). *Bifidobacterium* is considered to be the most important probiotic bacteria in humans (39). Tan et al. (19) found that the dysbiosis of gut microbiota in IgAN patients was characterized by a significant reduction in *Bifidobacterium*, which was negatively correlated to the level of proteinuria and hematuria. These indicated that the abundance of gut microbiota was related to the severity of IgAN. Additionally, some studies have also established random forest models based on gut microbiota to identify IgAN patients (22, 24, 25, 33). This suggested that gut microbiota has the potential to be a biomarker for the diagnosis of IgAN. Studies comparing the gut microbiota of IgAN patients with those of normal people are summarized in **Table 2**. The major intestinal dysbiosis in IgAN is depicted in **Figure 1**.

**Table 2:** The alteration of gut microbiota in IgAN patients.

Study(Year)	Human Subjects(n)	Region	The alteration of gut microbiota	Correlation	Reference (PMID)
De Angelis,et al. (2014) (29)	IgAN (30) vs. HC (15)	Italy	**Phylum:** Firmicutes↑	–	24922509
			**Family:** Bacteroidaceae and Prevotellaceae↓; Bifidobacteriaceae↓; Coriobacteriaceae↑		
			**Species:** Sutterellaceae and Enterobacteriaceae↑; Bifidobacterium↓		
Hu, et al. (2020) (33)	IgAN (17) vs. HC (18)	Hunan, China	**Phylum:** Firmicutes↑; Fusobacteria↑; Bacteroidetes and Proteobacteria↓; Synergistetes↓	Escherichia-Shigella was negatively associated with the estimated glomerular filtration rate (eGFR) but was positively associated with the urinary albumin-to-creatinine ratio.	32169051
			**Family:** Proteobacteria↑; Enterobacteriaceae ↑; Synergistaceae↓		
			**Genus:** Escherichia-Shigella↑; Hungatella↑; Eggerthella↑		
Zhong, et al. (2020) (26)	IgAN (52) vs. HC (25)	Sichuan, China	**Phylum:** Bacteroidetes↑; Firmicutes↓; Fusobacteria↑	Prevotella 7 and Bifidobacterium spp. were negatively correlated with Gd-IgA1. Escherichia-Shigella levels were negatively correlated with Prevotella 7.	33068859
			**Genus:** Bacteroides↑; Subdoligranula↓; Escherichia-Shigella↑; Lachnoclostridium↑; Blautia↓; Subdoligranulum and Prevotella 9 ↓; genus_Eubacterium hallii and Bifidobacterium↓		
Dong, et al. (2020) (34)	IgAN (44) vs. HC (30)	Xi'an, China	**Phylum:** Firmicutes↓; Proteobacteria ↑; Candidate_division_TM7↑; Fusobacteria↓; Synergistetes↓	Prevotella was positively correlated, while Klebsiella, Citrobacter, and Fusobacterium were negatively correlated with the level of serum albumin.	33194798
			**Genus:** Escherichia-Shigella↑; Defluviitaleaceae_incertae_sedis↑		
			Roseburia and Lachnospiraceae_unclassified ↓; Clostridium_sensu_stricto_1 and Fusobacterium↓		
He, et al. (2021) (30)	IgAN (119) vs. HC (45)	Beijing, China	**Family: **Erysipelotrichaceae↑	The decreased abundance of Dialister was associated with higher serum levels of Gd-IgA1.	33436510
			**Genus:** Dialister↓		
Wu, et al. (2021) (20)	IgAN (15) vs. HC (30)	Shenzhen, China	**Phylum:** Proteobacteria↑; Bacteroidetes↓	**-**	33553325
			**Genus:** Blautia↑; Bacteroides and Faecalibacterium↓; Streptococcus and Enterococcus ↑		
Sugurmar, et al. (2021) (28)	IgAN (36) vs. HC (12)	Malaysia	**Phylum:** Fusobacteria↑; Euryarchaoeota↓	**-**	33882859
			**Class:** Methanobacteria↓; Fusobacteriia ↑; Epsilonproteobacteria↑		
Chai, et al. (2021) (31)	IgAN (29) vs. HC (29)	Ningbo, China	**Phylum:** Firmicutes, Proteobacteria and Actinobacteria↑; Bacteroidetes↓	Acetic acid was positively associated with c_Clostridia, o_Clostridiales and g_Eubacterium_coprostanoligenes_group. Butyric acid was positively associated with g_Alistipes.	34082732
Zhang, et al. (2021) (32)	Male ESRD-IgAN (11) vs. male HC (7)	Guangzhou, China	**Male:** **Species:** Ruminococcus gnavus, Ruminococcus sp., Eubacterium dolichum, Bacteroides ovatus, and Phascolarctobacterium sp.↑; Megamonas sp., Roseburia sp., and Eubacterium biforme↓	Urine output, lymphocyte ratio, serum albumin, blood calcium, dialysis status, serum urea nitrogen, urine protein, and diabetes significantly correlated with fecal microbiota composition of male patients, whereas creatinine and 2-h post-prandial blood glucose significantly correlated with fecal microbiota composition of female patients.	34899638
	Female ESRD-IgAN (10) vs. female HC (8)		**Female:** **Species:** Ruminococcus gnavus, Ruminococcus sp., Eubacterium dolichum, Bacteroides ovatus, and Phascolarctobacterium sp.↑; Bacteroides ovatus, Prevotella copri, Roseburia faecis, Lachnospiraceae sp., and Roseburia sp.↓		
Shah, et al. (2021) (35)	progressive IgAN (20) vs. HC (20)	America	**Family:** Bacteroidaceae↑		35369657
			**Genus:** Escherichia-Shigella↑; Bacteroides↑; Prevotella 9↓		
Tang, et al. (2022) (27)	IgAN (35) vs. HC (20)	Shanghai, China	**Phylum:** Bacterioidetes↑; Proteobacteria↑; Fusobacteria↑; Firmicute↓; Actinobacteria↓	Actinobacteria, Bifidobacterium, Bifidobacteriaceae, and Bifidobacteriales were negatively and significantly correlated with urine Gd-IgA1 and the levels of intestinal mucosal barrier injury indexes. Shigella was positively correlated with the level of intestinal mucosal barrier injury indexes.	35872757
			**Genus:** Bacteroides↑; Faecalibacterium↑; Shigella↑; Bifidobacterium↓; Blautia↓		
Tan, et al. (2022) (19)	IgAN (35) vs. HC (25)	Sichuan, China	**Genus:** Bifidobacterium and Prevotella 9 ↓; Bacteroides↑	The abundance of Bifidobacterium was negatively related to urine protein levels and urine red blood cells. Bacteroides was positively correlated with urine protein levels and urine red blood cells. Prevotella 9 and urine red blood cells had a negative correlation.	36038927
Zhao, et al. (2022) (24)	IgAN (84) vs. HC (84)	China	**Phylum:** Proteobacteria↓	The abundance of Escherichia-Shigella was positively correlated with serum creatinine, proteinuria and the severity of Oxford pathologic classification and was negatively correlated with albumin and eGFR.	36041791
			**Genus:** Escherichia-Shigella↑; Pseudomonas↑; Erysipelatoclostridium↑; Lachnospira↓; Fusicatenibacter↓; Agathobacter↓; Romboutsia↓		
Liang, et al. (2022) (21)	IgAN (20) vs. HC (20)	Guangzhou, China	**Phylum:**Bacteroidetes↑; Firmicutes and Proteobacteria↓	Eggerthella lenta and Ruminococcus bromii were positively correlated with urine protein-creatinine ratio. Ruminococcus gnavus showed a direct association with red blood cells in urine. Bacteroides vulgatus, Ruminococcus gnavus, Clostridium bolteae and Tyzzerella nexilis were positively correlated with eGFR.	36090029
			**Class:** Bacteroidia and Negativicutes↑		
			**Order:** Bacteroidales and Burkholderiales↑; Clostridiales and Enterobacterales↓		
			**Family:** Tannerrellaceae↑; Ruminococcaceae↓; Eubacteriaceae↓		
			**Genus:** Bacteroides↑; Prevotella and Alistipesx↓		
			**Species:** Bacteroides plebeius and Bacteroides vulgatus↑; Prevotella copri and Alistipes putredinis↓		
Dong, et al. (2022) (22)	IgAN (117) vs. HC (150)	Zhengzhou, China	**Phylum:** Proteobacteria, Actinobacteriota↑; Patescibacteria ↑	Bifidobacterium was positively correlated with serum albumin. l-tryptophan, blood urea nitrogen and Eubacterium coprostanoligenes were positively correlated with each other.	36569195
			**Genus:** Escherichia-Shigella and Subdoligranulum ↑; Bifidobacterium and Dorea↑		
Hu, et al. (2022) (36)	Not available	Not available	**Genus:** Prevotella, Lachnospiracea_incertae_sedis and Megamonas↓; Escherichia_Shigella↑	–	36590596
Bao, et al. (2023) (23)	IgAN (19) vs. HC (15)	Beijing, China	**Phylum:** Bacteroidetes↓; Actinobacteria↑	Bifidobacterium was positively correlated with serum IgA levels, 24-hour urinary protein levels and the presence of hypertension. Escherichia-Shigella was positively correlated with urine red blood cells account.	36718700
			**Genus:** Escherichia-Shigella, Bifidobacte-rium, Dorea↑; Lachnospira, Coprococcus_2 and Sutterella↓		
Tang, et al. (2023) (25)	IgAN (25) vs. HC (20)	China	**Phylum:** Firmicutes↓; Bacteroidetes ↑; Proteobacteria↑; Fusobacteria↑; Actinobacteria↓	Coprococcus, Dorea, Bifidobacterium, Blautia, and Lactococcus were inversely associated with urinary Gd-IgA1 levels.	36878203
			**Genus:** Bacteroides↑; Faecalibacterium↑; Ruminococcus↑; Shigella↑; Bifidobacterium↓; Blautia↓; Roseburia↓; Coprococcus↓		

↑, higher abundance; ↓, lower abundance.

The aforementioned studies suggested that gut microbiota dysbiosis is present in patients with IgAN, but they are observational studies and therefore cannot establish a causal relationship between gut microbiota dysbiosis and IgAN. Recently, two Mendelian randomization studies both revealed the causal relationship between gut microbiota and IgAN (40, 41). However, there is a difference between two studies results. Ren et al. found (40) that *Enterorhabdus* is a protective factor for IgAN and *Butyricicoccus* is a risk factor for IgAN, while Wang et al. found (41) that *Actinobacteria* is associated with a high risk of IgAN, which may be due to differences in the way the two studies selected instrumental variables.

To sum up, IgAN patients showed gut microbiota dysbiosis, characterized by an increase in pathogenic bacteria and a decrease in beneficial bacteria. The dysbiosis of gut microbiota may be involved in the pathogenesis of IgAN. And the gut microbiota is expected to be a biomarker for the diagnosis of IgAN.

## Potential pathogenic role of gut microbiota in IgAN

### Disruption of the intestinal barrier

Normal gut microbiota plays an important role in maintaining the integrity of the intestinal mucosa. The destruction of the intestinal mucosal barrier is involved in the pathogenesis of IgAN (42). Increased intestinal permeability was found in both IgAN animal models and patients. Peng et al. (43) reported that IgAN rats had decreased intestinal epithelial tight junction protein expression and increased intestinal permeability. Serum levels of intestinal permeability markers were elevated in pseudosterile IgAN mice, which indicated that dysbiosis of gut microbiota might contribute to the disruption of intestinal mucosal barrier (25). Intestinal permeability was increased in mice induced by *Lactobacillus casei* cell wall extract and increased IgA in serum and IgA deposition in glomeruli were associated with intestinal permeability (44). Similarly, intestinal permeability was also found to be increased in IgAN patients. Tang et al. (27) also found that the indexes of intestinal mucosal barrier injury were higher in IgAN patients compared with normal subjects and positively related to the levels of Gd-IgA1 both in serum and urine. Furthermore, some genera of gut microbiota were correlated with intestinal mucosal barrier injury indexes (27). These suggest that intestinal mucosal destruction of IgAN may be related to dysbiosis of gut microbiota. In the case of damage to the intestinal mucosal barrier, toxic metabolites enter the blood circulation and produce an inflammatory response. P-cresol sulfate and indole sulfate can stimulate inflammation by promoting the release of pro-inflammatory cytokines, which is related to IgAN (45, 46).

### Changes in metabolites associated with gut microbiota

Gut microbiota may also be involved in the development and progression of IgAN through altered metabolites. It was found that microbial metabolic pathways altered in IgAN, and the alteration of microbial metabolic pathways might be triggered by the alteration of gut microbiota (18). Wu et al. (20) reported that IgAN patients had different serum and fecal metabolites from normal people, and these metabolites had a correlation with gut microbiota. Hard-to-digest carbohydrates in food are fermented by anaerobic colonic bacteria to produce short-chain fatty acids (SCFAs), which mainly include acetate, propionate and butyrate (47). Chai et al. (31) found that levels of SCFAs including acetic acid, butyric acid, caproic acid, isobutyric acid, and propionic acid were significantly lower in IgAN patients compared with normal controls. And caproic acid was negatively correlated with 24-hour urinary protein levels (31). Interestingly, supplementation of IgAN mice with SCFAs reduced IgA renal deposition, improved mesangial hyperplasia and decreased urinary protein levels, which was achieved by the inhibition of NLRP3/ASC/Caspase 1 signaling pathway (19). In addition, Wu et al. (20) reported that the changes of gut microbiota in IgAN affected the metabolism and absorption of polyunsaturated fatty acids associated with gut microbiota. Long-chain n-3 polyunsaturated fatty acid in fish oil can inhibit the IgAN induced by trichothecene mycotoxin deoxynivalenol in mice (48). Dysbiosis of gut microbiota causes dysregulation of linoleic acid and arachidonic acid metabolism, reduction of prostaglandin derivatives, and imbalance between anti-inflammatory and pro-inflammatory fatty acids, which are associated with impaired intestinal mucosal barrier (49).

In addition to lipid metabolism disorders, there are also amino acid metabolism disorders in intestinal metabolism of IgAN patients. In amino acid metabolism disorder, aromatic tryptophan metabolism disorder is the most remarkable (49). The serum level of l-tryptophan is higher in IgAN patients, and it was positively correlated with blood urea nitrogen, serum creatinine and 24 total urinary protein, but negatively correlated with the eGFR (22). Interestingly, l-tryptophan is positively correlated with *Eubacterium-coprostanoligenes* (22). However, the gut-derived metabolite of tryptophan 3-indolepropionic acid significantly decreases in the gut and blood, which is believed to be related to decreased levels of intestinal *Bacteroidetes* (49). Furthermore, 3-indolepropionic acid is the primary ligand of the aryl hydrocarbon receptors (AhR) signaling and an important part of the intestinal mucosal immune barrier, promoting the renewal of the intestinal mucosal epithelium and maintaining the integrity of the intestinal mucosa (50). 3-indolepropionic acid was negatively correlated with Gd-IgA1 serum and positively correlated with eGFR (49). In addition, the serum B cell activating factor of the tumor-necrosis factor family (BAFF) levels were significantly higher in IgAN patients, and the five bacterial metabolites of 4-(1,1,3,3-tetramethylbutyl) phenol, p-tert-butyl-phenol, methyl neopentyl phthalic acid, hexadecyl ester benzoic acid and furanone A were positively correlated with serum BAFF levels (51).

In brief, the changes of metabolites caused by gut microbiota dysbiosis might be involved in the pathogenesis and progression of IgAN.

### Aberrant mucosal immune response

Excessive production of aberrantly glycosylated IgA1 is considered to be the causative factor of IgAN (52). It was found that the glomeruli of IgAN patients had abundant deposition of these aberrantly glycosylated IgA1, also called Gd-IgA1 (53). However, the source of Gd-IgA1 is not fully understood. Some studies showed that gut-associated lymphoid tissue (GALT) is involved in the production of Gd-IgA1 (54, 55). Under normal circumstances, gut microbiota, intestinal adaptive immune cells, and secretory immunoglobulin A coordinate with each other to maintain tolerance to symbiotic bacteria and defense against invading pathogens, thereby maintaining intestinal homeostasis (56). Alterations of the gut microbiota in IgAN may disrupt this homeostasis and lead to intestinal microbial infections. Toll-like receptors (TLRs), regarded as recognizers of pathogen-associated molecular patterns, play an important role in sensing microbial infection and participate in the pathogenesis of IgAN (54). Long-term and excessive activation of TLRs eventually leads to the overproduction of IgA1/Gd-IgA1 (57). Intestinal microbial infections boost the class switch of naive B lymphocytes to IgA antibody-secreting cells by T cell-independent and T cell-dependent pathways (58). BAFF and a proliferation-inducing ligand (APRIL), which are thought to be secreted by gut macrophage-dendritic cells and stromal cells, are involved in the T cell-independent pathway thereby promoting class switching of B cells from IgM to IgA (58). Due to abnormal homing, the lymphoplasmic cells producing mucosal IgA1 gradually migrate from mucosal lymphoid tissue to bone marrow, which leads to excessive production of mucosal-type IgA1 in the systemic circulation (59). Increased IgA1 complexes resulted in the shedding of IgA Fc receptor called CD89 from myeloid cell surfaces and integrated the soluble part of CD89 into complexes, which then deposits in kidney mesangium (60, 61).

The speculation that gut microbiota is involved in the aberrant intestinal mucosal immune response in IgAN is supported by the following studies. BAFF overexpressing transgenic mice exhibited IgAN manifestations including high circulating levels of abnormally glycosylated polymerized IgA and renal deposition of IgA exclusively in the presence of gut microbiota, and pathological manifestations of IgAN were not seen in germ-free mice (62, 63). In a specific pathogen-free environment, a1K1 mice had less mesangial IgA deposition than in conventional environments (64). Transplantation of gut microbiota from patients with progressive IgAN to humanized IgAN mice resulted in increased serum BAFF and Gd-IgA1 and decreased surface CD89 expression on blood CD11b+ cells which was related to mesangial deposition of soluble CD89 and IgA1 (65). Multiple intraperitoneal injections of *Lactobacillus casei* cell wall extract resulted in an increase in circulating IgA and IgA-IgG complexes and deposition of IgA in the glomerular mesangium in mice (44). Tang et al. (25) found that the levels of BAFF and APRIL were increased in the intestinal tissue of IgAN mouse model and revealed that dysbiosis of gut microbiota might contribute to local immune responses in IgAN. In addition, the abundance of *Bifidobacterium* in IgAN patients is lower than that in healthy controls (19, 26, 27). *Bifidobacterium* was found to have a negative correlation with serum Gd-IgA1 (26). *Bifidobacterium* was negatively correlated with urine Gd-IgA1 which had greater value in the diagnosis of IgAN (25, 27). The gut microbiota of IgAN patients was rich in *Flavonifractor plautii*, which can secrete α-galactosidase and α-N-acetyl-galactosaminidase that might be associated with the production of Gd-IgA1 (21). IgAN patients had an increased abundance of mucin-degrading bacteria including *Akkermansia muciniphilan* which can deglycosylate IgA1 and make it be recognized by autoreactive IgG in the serum of patients with IgAN (66). Mice expressing human IgA1 and human FC-α receptors developed an aggravated IgAN phenotype under intestinal colonization by *Akkermansia muciniphilan*, which indicated that gut microbiota dysbiosis contributes to the generation of auto-antigens in IgAN (66). Furthermore, mice colonized with gut microbiota from IgAN patients showed the IgAN phenotype with the activation of TLR4/MyD88/NF-κB pathway and B-cell stimulators (67). In peripheral blood mononuclear cells, lipopolysaccharide activated TLR4/MyD88/NF-κB pathway, B-cell stimulators, and proinflammatory cytokines and resulted in the overproduction of Gd-IgA1, which suggested gut dysbiosis can stimulate the overproduction of Gd-IgA1 through TLR4 signaling pathway (67). Similarly, Tang et al. (68) also revealed that gut dysbiosis mediated renal injury in IgAN by activating TLR4 signaling pathway. Interestingly, in recent years, Nefecon, a novel budesonide capsule designed to be released in the distal ileum, targeting to the mucosal immune system, has been found to delay the decline of eGFR and lastingly reduce proteinuria in IgAN patients in a phase III clinical trial (69).

In a word, gut microbiota dysbiosis leads to intestinal microbial infection, which in turn leads to aberrant immune responses causing the increase of Gd-IgA1 in the circulation, and the deposition of immune complexes containing Gd-IgA1 in the glomerular mesangium results in glomerular injury. The aberrant immune responses caused by the alterations in the gut microbiota are summarized in **Figure 1**.

### Potential therapies for IgAN by modulating gut microbiota

Based on the possible involvement of gut microbiota in the pathogenesis of IgAN, more and more studies have attempted to treat IgAN by modulating gut microbiota. Fecal microbiota transplantation can modulate the composition of gut microbiota in IgAN mice. After receiving gut microbiota from healthy people, humanized IgAN mice exhibited decreased proteinuria, increased blood CD11b+ cell surface CD89 expression, and decreased expression of KC chemokine in the kidney, which is found to stimulate the proliferation of renal epithelial cells (65). Zhen Wu Tang, a classic Chinese herbal prescription, has been widely used to treat various kidney diseases in clinics in China for more than one thousand years for effectively relieving edema, dysuria, and oliguria symptoms (70, 71). A recent study found that treatment of Zhen Wu Tang made the gut microbiota of IgAN rats tend to approximate that of normal rats, modulated the metabolicphenotype perturbation, reversed morphological changes including glomerulus swelling, proliferation of mesangial cells and extension of mesangial matrix, decreased inflammation of intestinal mucosa and reduced kidney damage in IgAN rats (18). Probiotics have been used in the treatment of diarrhea, inflammatory bowel disease, irritable bowel syndrome and other diseases with certain efficacy and safety (72). Interestingly, probiotic supplementation has also been found to improve IgAN. Soylu et al. found (73) that *Saccharomyces boulardii* could prevent IgAN induced by oral poliovirus vaccine in mice, which may be achieved by downregulating systemic IgA response to the stimulation of enteral antigenic. Treatment of probiotics containing *Bifidobacterium* relieved gut microbiota dysbiosis in IgAN mice through increasing beneficial bacteria and decreasing potentially pathogenic bacteria and alleviated the IgAN manifestations in mouse models by blunting the NLRP3 activating signal (19). In addition, the treatment of antibiotics depleted the gut microbiota of IgAN mice and impaired GALT architecture, significantly preventing human immunoglobulin A1 deposition in mesangium, glomerular inflammation and proteinuria (74). Rifaximin is thought to promote the growth of probiotics, prevent intestinal inflammation, and regulate intestinal barrier function (75, 76). IgAN mice treated with antibiotic rifaximin showed decreased urinary protein, serum hIgA1-sCD89 and mIgG-hIgA1 complex levels, gut expression levels of BAFF, human IgA1 glomerular deposition and CD11b+ cell infiltration (77). Hydroxychloroquine treatment diminished abnormal glycosylation levels in IgAN through activating the C1GALT1/Cosmc pathway, ameliorated renal tissue pathological damage, and mitigated intestinal permeability and the dysbiosis of gut microbiota in IgAN rats (78). Gluten is a key protein found in wheat, barley and rye, and the gluten-free diet can affect the composition of human gut microbiota (79). Papista et al. (80) found that a gluten-free diet prevented the development of IgAN in α1KI-CD89Tg mice.

The treatment of modulating the gut microbiota has not only played a role in IgAN animal models but also achieved certain effects in IgAN patients. Zhao et al. (81) performed the first fecal microbiota transplantation to IgAN patients and found that feces from healthy people modulate the gut microbiota of two patients with refractory IgAN, resulting in decreased 24-hour urinary protein, elevated serum albumin, and stable renal function. After fecal microbiota transplantation, urinary protein had a significant decrease and even turned negative in one IgAN patient who refused to take immunosuppressive drugs (82).

Although the methods mentioned above for modulating the gut microbiota all seem to improve IgAN, long-term use of broad-spectrum antibiotics comes with inevitable side effects. Fecal microbiota transplantation appears to be the most powerful method for modulating the gut microbiota, and it has become an effective treatment for recurrent *Clostridium difficile* infections (83). However, the long-term effects of fecal microbiota transplantation are still unknown. Furthermore, treatment of IgAN by fecal microbiota transplantation is also performed only in individual populations currently.

**Figure 2:**
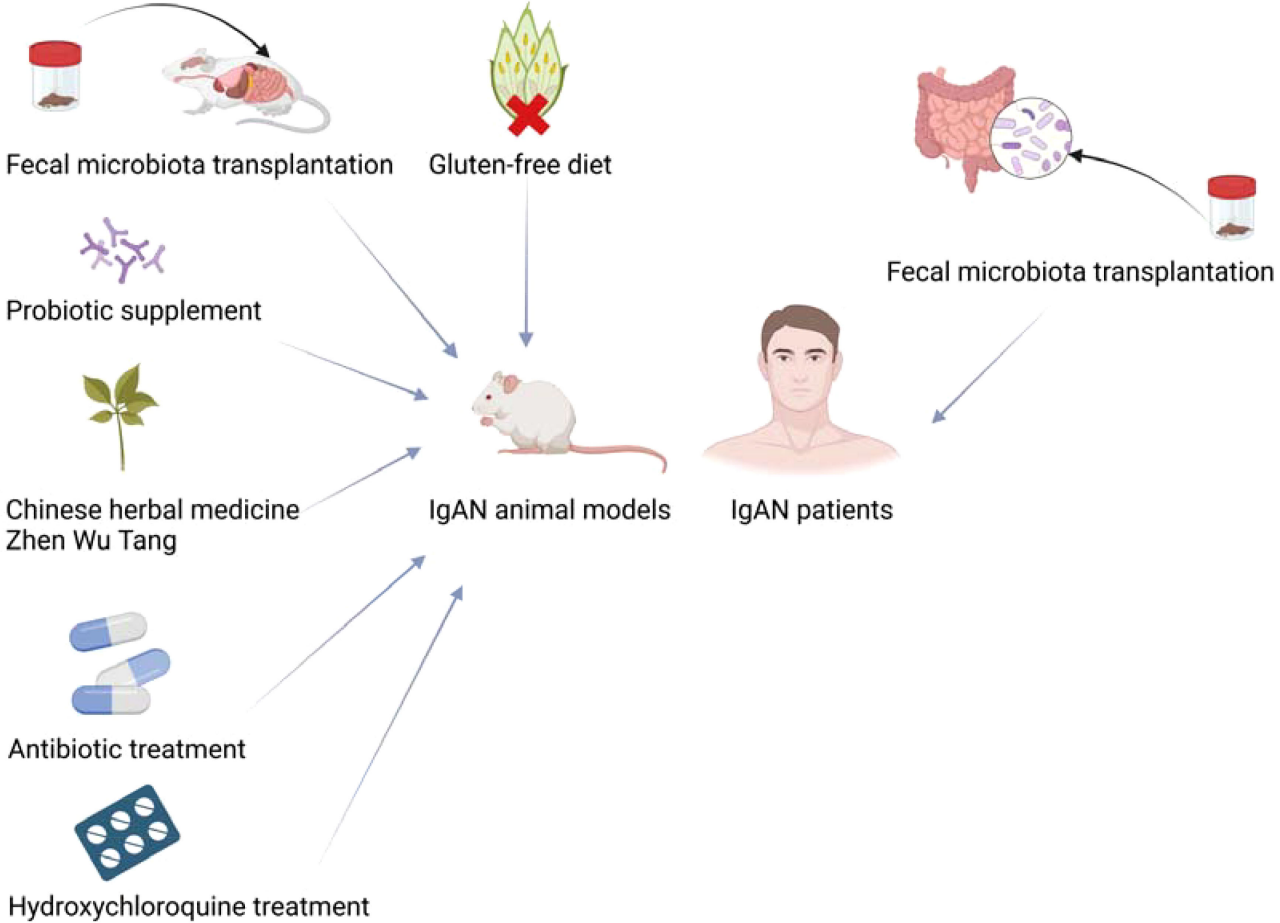
Modulation of gut microbiota treats IgAN animal models and patients. Created with BioRender.com.

In summary, gut microbiota is a potential target for the treatment of IgAN, and the treatments by modulating gut microbiota are expected to become a new therapy for IgAN. Current therapeutic approaches to try to treat IgAN by modulating the gut microbiota are shown in **Figure 2**.

## Conclusion

Gut microbiota dysbiosis is present in IgAN, which is characterized by a decrease in beneficial bacteria and an increase in potentially pathogenic bacteria. Gut microbiota may be involved in the development and progression of IgAN through the destruction of intestinal barrier, altered metabolites and aberrant mucosal immune responses. Gut microbiota may be a potential biomarker for the diagnosis of IgAN and a potential target for the treatment of IgAN. Therapies through modulating gut microbiota have effects on the improvement of IgAN. However, attempts to treat IgAN by modulating gut microbiota have mostly only been implemented in animal models now. Large-scale clinical trials are needed to verify the efficacy and safety of methods for modulating gut microbiota in the treatment of IgAN.
